# Incidents in the context of pre-hospital care by ambulances: contributions to patient safety

**DOI:** 10.1590/0034-7167-2022-0657

**Published:** 2023-11-27

**Authors:** Eric Rosa Pereira, Graciele Oroski Paes

**Affiliations:** I Faculdades Souza Marques. Rio de Janeiro, Rio de Janeiro, Brazil; II Universidade Federal do Rio de Janeiro. Rio de Janeiro, Rio de Janeiro, Brazil

**Keywords:** Patient Safety, Emergency Medical Services, Ambulances, Prehospital Care, Universal Health Care, Seguridad del Paciente, Servicios Médicos de Urgencia, Ambulancias, Atención Prehospitalaria, Atención de Salud Universal, Segurança do Paciente, Serviços Médicos de Emergência, Ambulâncias, Assistência Pré-Hospitalar, Assistência de Saúde Universal.

## Abstract

**Objectives:**

to analyze the occurrence of incidents in the context of mobile terrestrial pre-hospital care.

**Methods:**

a descriptive research was carried out through the observation of 239 treatments performed by 22 healthcare professionals at the Mobile Emergency Care Service, located in Baixada Fluminense, Rio de Janeiro, Brazil. Fisher’s exact test and chi-square test were used for data analysis.

**Results:**

the total time dedicated to patient care was 439.5 hours, during which 2386 security incidents were observed. The most notable ones were related to written communication (235), patient identification through bracelets (238), and safety in medication preparation (81).

**Conclusions:**

the need to promote and implement initiatives aimed at patient safety is evident, with special focus on international safety goals within the scope of mobile pre-hospital care services.

## RESUMO

**Objetivos:**

analisar a ocorrência de incidentes no contexto do atendimento pré-hospitalar móvel terrestre.

**Métodos:**

realizou-se uma pesquisa descritiva através da observação de 239 atendimentos efetuados por 22 profissionais de saúde no Serviço de Atendimento Móvel de Urgência (SAMU), situado na Baixada Fluminense, Rio de Janeiro, Brasil. Para a análise dos dados, empregaram-se o teste exato de Fisher e o teste qui-quadrado.

**Resultados:**

o tempo total dedicado ao atendimento ao paciente foi de 439,5 horas, durante o qual se observaram 2386 incidentes de segurança. Os mais notáveis foram relativos à comunicação escrita (235), identificação do paciente através de pulseiras (238) e à segurança na preparação de medicamentos (81).

**Conclusões:**

evidencia-se a necessidade de promover e implementar iniciativas que visem a segurança do paciente, com foco especial nas metas internacionais de segurança, no âmbito dos serviços de atendimento pré-hospitalar móvel.

## RESUMEN

**Objetivos:**

analizar la ocurrencia de incidentes en el contexto de la atención prehospitalaria móvil terrestre.

**Métodos:**

se realizó una investigación descriptiva a través de la observación de 239 atenciones realizadas por 22 profesionales de la salud en el Servicio de Atención Móvil de Urgencia, ubicado en la Baixada Fluminense, Río de Janeiro, Brasil. Para el análisis de los datos, se emplearon la prueba exacta de Fisher y la prueba de chi-cuadrado.

**Resultados:**

el tiempo total dedicado a la atención al paciente fue de 439,5 horas, durante las cuales se observaron 2386 incidentes de seguridad. Los más destacados fueron relacionados con la comunicación escrita (235), la identificación del paciente a través de pulseras (238) y la seguridad en la preparación de medicamentos (81).

**Conclusiones:**

se evidencia la necesidad de promover e implementar iniciativas que apunten a la seguridad del paciente, con un enfoque especial en las metas internacionales de seguridad, en el ámbito de los servicios de atención prehospitalaria móvil.

## INTRODUCTION

In 2013, the Brazilian Ministry of Health launched the National Patient Safety Program (NPSP), established by GM Ordinance No. 529 of April 1, 2013, aiming to address, among other things, the demand for the prevention of adverse events in health services. In the same year, Collegiate Board Resolution No. 36 proposed the need to create Patient Safety Centers in health services, but not necessarily in Mobile Pre-Hospital Care Services (MPHC)^(1)^.

Patient safety is understood as an organized structure of activities that create cultures, processes, procedures, behaviors, technologies, and environments in the healthcare area that consistently and sustainably reduce risks, decrease the occurrence of avoidable harm, make errors less likely, and reduce their impact when they occur^(2)^. Health incidents are defined as circumstances that have the potential to cause harm to the patient; when discernible harm occurs, it is called an adverse event^(3)^.

With the aim of minimizing these factors, the Brazilian Ministry of Health, in line with international patient safety goals, formulated care protocols focused on hand hygiene, effective communication, safe surgery, prevention of pressure injuries (added to the sixth international patient safety goal), falls, patient identification, and safety in prescription, use, and administration of medications^(4)^.

MPHC is characterized by emergency support provided outside the hospital setting, at the site of the accident or where emergency care is needed, with the purpose of providing fast and accurate assistance, thereby increasing the patient’s chances of survival^(5)^. However, more than providing skilled and swift care, it is necessary to deliver it safely, based on evidence-informed clinical decisions to maximize expected outcomes and minimize or eliminate potential patient harm^(6)^.

Due to its high-risk nature, the commitment of regulatory and supervisory agencies is necessary to formalize norms that standardize and regulate safety actions in healthcare in this context. Studies addressing health incidents in MPHC are scarce, including in Brazil, as it is not possible to generalize the frequency and types of adverse events in hospitals to the pre-hospital context^(7)^, given that the scenario is dynamic and sometimes uncontrollable. In this environment, patient safety is less known and investigated, often due to operational pressures, such as the urgency of care due to the patient’s severity and the difficulty of transferring between pre-hospital and intra-hospital services.

In MPHC, studies indicate a propensity for adverse events related to medication administration, use of technologies, patient assessment, and hand hygiene, characterizing unsafe scenes with a risk of additional injuries resulting from transportation or inadequate care^(3,8)^.

Thus, concern about safety in MPHC arises from the high number of incidents during healthcare, highlighting failures related to clinical procedures performed during care, documentation of care records, administration of intravenous fluids, use of oxygen therapy, faulty medical equipment, inadequate infrastructure, scarce human and material resources, and non-adherence to institutional protocols^(6,9)^.

In addition to the actions of the Brazilian Ministry of Health, the World Health Organization (WHO) considers ambulance care as a healthcare facility that needs measures to minimize patient safety risks, as presented in the Global Action Plan to eliminate preventable harm in healthcare from 2021 to 2030^(2)^. The plan emphasizes the need to prioritize patient safety-related research in high, middle, and low-income countries, as well as capacity-building projects for the teams involved. The identification, development, and promotion of interventions worldwide to improve patient safety are encouraged.

This research is justified since healthcare is provided wherever individuals require health interventions. However, although the public ambulance service is present in much of the Brazilian territory, little is known about the safety risks in this setting.

## OBJECTIVES

To analyze the occurrence of incidents during mobile pre-hospital care (MPHC).

## METHODS

### Ethical Aspects

This study is the result of a master’s dissertation entitled “Mapping safety incidents in mobile pre-hospital care: contributions to healthcare practice,” which was submitted and approved by the Ethics Committee of Hospital Escola São Francisco de Assis/Escola de Enfermagem Anna Nery/Universidade Federal do Rio de Janeiro. The research began after approval from the mentioned Ethics Committee, and the participants who agreed to take part in the study signed the Informed Consent Form (ICF), which was provided in duplicate: one copy for the researcher and another for the participant.

### Design, Study Location, and Period

This is a descriptive and observational study, constructed following the Strengthening the Reporting of Observational Studies in Epidemiology (STROBE) guidelines. The setting was the Mobile Emergency Care Service (Portuguese acronym: SAMU) in a municipality in Baixada Fluminense, in the metropolitan region of Rio de Janeiro. The observation period took place between July 2018 and February 2019, with one researcher present during each assistance. The chosen method was non-participant observation, meaning the researcher did not directly or indirectly interfere in the assistance provided during the study.

### Sample, Inclusion, and Exclusion Criteria

The participants in the research were 22 healthcare professionals, including 14 nursing technicians, seven nurses, and one doctor, who worked in ambulance care. The selection of participants followed inclusion criteria, which required a minimum of six months of experience in the respective roles, and participants on medical leave were excluded.

For sample calculation, the parameters of the monthly average of care over a 12-month period were considered, with 210 monthly basic ambulance care (for patients with life risk not classified as potentially needing medical intervention on-site and/or during transportation) and 64 monthly advanced ambulance care (for high-complexity patients requiring intensive medical care). A 95% confidence level and 5% maximum sample error were established for the research. The total sample comprised 239 incidents, with 105 in basic ambulance care and 134 in advanced ambulance care. The inclusion criteria were terrestrial pre-hospital care in clinical, psychiatric, pediatric, obstetric, and traumatic emergencies at homes or public places. The exclusion criteria were occurrences for inter-hospital transfers.

### Study Protocol

A structured observation script was used, constructed from the incident classification manual described by the WHO^(3)^. The instrument focused on the Dimension of Direct Patient Care, comprising 54 variables grouped into 9 blocks related to Clinical Administration, Clinical Process and Procedure, Documentation, Healthcare-Associated Infection, Medication and Intravenous Fluids, Oxygen/Gas/Vapor, Medical Devices/Equipment, and Patient Accidents/Falls. This study addressed variables related to international patient safety goals^(3)^. The safe surgery goal was excluded as it does not apply to the context under investigation.

The first block of victim care was named “Patient Data,” consisting of 12 variables coded from A1 to A12. This block included continuous quantitative variables: a) date of care; and b) time of ambulance team activation by the control center, departure from the base for care, arrival at the location of care, departure from the location of care, arrival at the destination hospital, and return to the base. Additionally, it included nominal quantitative variables: a) type of ambulance for care; b) gender; c) type of care (psychiatric, clinical, traumatic, obstetric, and pediatric); and d) age.

The second block aimed to verify variables related to patient safety, considering the Brazilian protocol^(3)^ and international patient safety goals. Each item had response options: “yes,” “no,” or “not applicable” as alternatives applied to its observation. The observations followed the following variables: Patient Identification; complete filling of the care form; legibility of information in the care form; handover of the occurrence to the hospital service; medication prescription before or after administration; proper preparation of medication; dosage according to the prescription; existence of proper space for medication storage; hand hygiene between procedures; change of gloves after each procedure; peripheral venous puncture performed in an aseptic manner; aseptic urinary catheterization; washing of wounds with saline before applying the dressing; use of immobilization equipment; use of immobilizers (head straps and seatbelts) during care; occurrence of tripping, slipping, falling, or loss of patient balance; verification of contact between the victim’s skin and the equipment used; and occurrence of patient accidents involving scratches, cuts, punctures, or penetrations.

### Analysis of Results and Statistics

For data treatment, the statistical programs used were Epi Info version 7.2 and Statistical Package for Social Science (SPSS) version 25.0.0.0. The analysis was performed using descriptive and inferential measures. Fisher’s exact test and chi-square (x^2^) were applied to verify the association between qualitative variables. A significance level of 5% was adopted for all tests. The variables were expressed through absolute and relative frequencies.

## RESULTS

The results will be presented as follows: characteristics of patient care and mapping of incidents related to the Brazilian national protocol and international patient safety goals applicable to MPHC, with the exception of Safe Surgery.

The total direct patient care time amounted to 439.5 hours of observation, during which 2386 incidents were identified. Among these, 708 incidents did not align with the Brazilian national protocol and international patient safety goals, while 1678 were related, as follows: 794 incidents related to effective communication, 383 incidents associated with hand hygiene and infection risk, 237 incidents related to correct identification, 192 incidents related to medication safety, and 72 incidents related to the risk of falls and pressure injuries.

Table 1 presents the characteristics of patient care. It can be observed that the mean age of patients was 50.3 years, with a standard deviation of 22.5 years. The most significant occurrence was clinical emergencies (n=137; 57.3%), followed by traumatic emergencies (n=57; 23.8%), psychiatric emergencies (n=27; 11.3%), and obstetric emergencies (n=17; 7.1%). Regarding gender, males (n=133; 56.4%) prevailed compared to females (n=103; 43.6%).

**Table 1:** Description of variables related to care in Basic and Advanced Ambulances, Rio de Janeiro, Rio de Janeiro, Brazil, 2019

Characteristics	n(%)	Mean/SD
Age of patients attended		50.3/22.5
Number of care instances		
Basic	105(43.9)	
Advanced	134(56.1)	
Type of care		
Psychiatric	27(11.3)	
Clinical	137(57.3)	
Traumatic	57(23.8)	
Obstetric	17(7.1)	
Gender of patients		
Male	133(56.4)	
Female	103(43.6)	

Table 2 presents characteristics related to the time between activation and completion of the occurrence. It can be observed that the average time between activation and departure from the base for care was 6 minutes, and the time between arrival at the patient (start of direct care) until the team’s departure from the hospital (complete transfer to the hospital) was 88 minutes.

**Table 2:** Evaluation of the average ambulance travel time during the observation period, Rio de Janeiro, Rio de Janeiro, Brazil, 2019

Studied characteristics	Mean	Standard Deviation	Minimum/Maximum values
Time between activation and departure from the base	6.1’	4.6	1-28
Time between departure from the base and arrival at the location	17.0’	13.6	2-90
Time between arrival at the location and departure from the scene	23.0’	14.1	2-90
Time between arrival at the location and departure from the destination hospital	88.7’	45.1	12-300
Total time of care	110.3’	62	15-325

*The time is considered in minutes (x’).*

**Figure 1:**
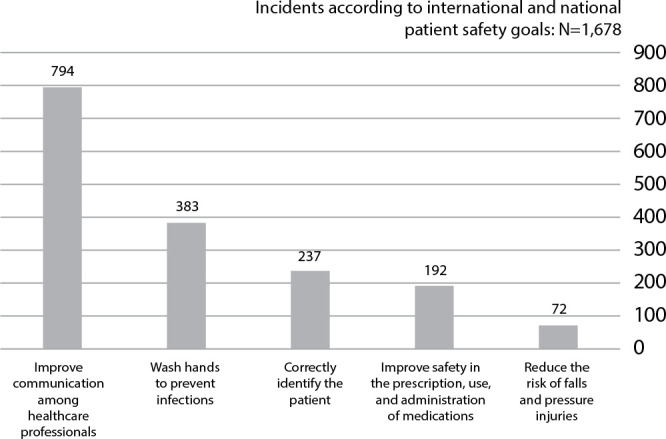
Total number of incidents according to international and national patient safety goals, Rio de Janeiro, Rio de Janeiro, Brazil, 2019

Figure 1 presents the incidents mapped based on international patient safety goals, according to WHO (2009) (3), and the Brazilian national protocol^(6)^. In this case, Goal number 4 - Safe Surgery, was excluded as it does not apply to MPHC.

Table 3 lists safety incidents according to international goals: Patient Identification, Effective Communication, Medication Safety, Hand Hygiene to Prevent Infections, Fall Prevention, and Pressure Injury Prevention.

**Table 3:** Association between compliance with patient safety goals and the type of ambulance used, Rio de Janeiro, Rio de Janeiro, Brazil, 2019

Characteristics related to patient safety goals:	n(%)	Vehicle Type		*p* value^*^
		Basic	Advanced	
		n(%)	n(%)	
Goal 1: Patient Identification				
Yes	-	-	-	-
No	238 (100)			
Goal 2: Effective Communication				
Completed the full care form				
Yes	4 (1.7)	-	4(3.0)	0.097
No	235(98.3)	105(100.0)	130(97.0)	
Information on the care form is legible				
Yes	111(46.4)	99(94.3)	12(9.0)	<0.001
No	128(53.6)	6(5.7)	122(91.0)	
Handover of the occurrence to the hospital service was done				
Yes	123(64.4)	58(81.7)	65(44.2)	<0.001
No	68(35.6)	13(18.3)	55(45.8)	
Goal 3: Medication Safety				
Medications administered were prescribed before or after administration				
Yes	124(94.7)	42(91.3)	82(96.5)	0.209
No	7 (5.3)	4(8.7)	3(3.5)	
Proper preparation of the medication was done				
Yes	50(38.2)	25(54.3)	25(29.4)	0.005
No	81(61.8)	21(45.7)	60(70.6)	
The dosage was according to the prescription				
Yes	126(99.2)	43(97.9)	83(100)	0.168
No	1(2.3)	1(2.3)	-	
There is storage space for the medication				
Yes	236(99.2)	103(98.1)	133(100.0)	0.194
No	2(0.8)	2(1.9)	-	
Goal 5: Infection Risks				
Hand hygiene was performed between procedures				
Yes	17 (7.2)	17(16.3)	-	<0.001
No	218(92.8)	87(83.7)	131(100.0)	
Gloves were changed after each procedure				
Yes	71(30.9)	41(41.4)	30(22.9)	0.003
No	159(69.1)	58(58.6)	101(77.1)	
Peripheral venous puncture was performed in an aseptic manner				
Yes	65(39.2)	31(50.8)	34(32.4)	0.019
No	101(60.8)	30(49.2)	71(67.6)	
Urinary catheterization was performed in an aseptic manner				
Yes	1(14.3)	1(25.0)	-	0.350
No	6 (85.7)	3(75.0)	3(100)	
Wound washing with saline was done before applying the dressing				
Yes	22(53.7)	9(64.3)	13(48.1)	0.326
No	19(46.3)	5(51.9)	14(51.9)	
Goal 6: Fall/Pressure Injury Prevention				
Used immobilization equipment				
Yes	185(77.7)	66(78.6)	119(95.2)	<0.001
No	24(10.0)	18(21.4)	6(4.8)	
Used immobilizers (head straps and seatbelts) during care				
Yes	150(63)	48(75.0)	102(83.6)	0.158
No	36(15.1)	16(25.0)	20(16.4)	
The patient tripped, slipped, fell, or lost balance				
Yes	12(5.0)	4(3.9)	8(6.1)	0.444
No	222(93.2)	99(96.1)	123(93.9)	
Checked contact between the victim's skin and the equipment used				
Yes	15(6.3)	6(8.6)	9(7.6)	0.817
No	173(72.6)	64(91.4)	109(92.4)	
Accidents occurred to the patient involving scratches, cuts, punctures, or penetrations				
Yes	2(0.8)	2(2.0)	-	0.182
No	227(95.3)	96(98.0)	131(100.0)	

* *Pearson's chi-square test or Fisher's exact test for variables with expected values <5; Significance level p<0.05.*

## DISCUSSION

The Brazilian SAMU is present in 82% of cities, covering over 3,500 municipalities. The ambulance distribution includes 2,702 basic support units and 605 advanced support units, considering that the highest demand for the service comes from patients with clinical comorbidities without immediate life risk^(10)^.

Regarding patient identification, there is also low adherence in hospital settings, but in significantly smaller proportions than in MPHC. The Ministry of Health recommends the use of a simple identification bracelet on the patient’s right wrist, containing at least two pieces of information: full name without abbreviations, complete address, date of birth, document registration, and the location where they were found^(4)^.

Among the errors associated with identification failures in hospitals are: blood and blood product administration, blood collection, sample collection for clinical exams, surgical procedures, radiological exams, and incorrect medication administration^(11)^.

Although not a regulated practice in MPHC, this study observed a significant number of cases where patients were not identified with an identification bracelet, both in basic support and advanced life support ambulances. It is recommended that patient identification with a bracelet be done as soon as they enter the ambulance. This task can be performed by any available team member, especially the rescue driver.

Failure to identify the patient during the initial care may put them at risk of receiving incorrect procedures. Thus, if the patient remains unidentified during their hospital stay, it may compromise their safety.

More than the use of identification bracelets, standardization is important to regulate their use and ensure the reduction of adverse events and incidents in healthcare. For this, the creation of protocols, as well as education and awareness actions for professionals, is important and necessary for adherence to their use^(12)^. A study conducted in Brazil^(8)^ suggests patient identification through bracelets with colors indicating severity. However, the authors do not address the need to identify patients with at least two pieces of information.

There is a significant delay regarding patient identification in MPHC. This challenge, as in the hospital environment, must be approached in a multidisciplinary manner, with the participation of all involved. To minimize healthcare risks, two approaches can be adopted in MPHC: the use of bracelets and/or adhesive labels. It is up to the institutions that provide MPHC to determine which process is more viable, as what should be avoided is a first healthcare encounter without proper patient identification.

Communication between pre-hospital and intra-hospital care teams was found to be weakened in both basic and advanced support. Effective communication among participants indicated the need for improvement, and the handover of the occurrence has not been adequately performed between the teams. In addition to this, incomplete filling of the patient care form emerged as an area in need of improvement. There are illegible pieces of information on these forms, with a significant number found among advanced life support teams.

Studies affirm that patient transfer plays a crucial role in care and has been extensively investigated in hospital settings. However, it receives less attention when it occurs between pre-hospital and intra-hospital care^(13)^. In Brazil, at the national level, there is no consensus on the use of a single specific protocol for patient transfer between the MPHC service and intra-hospital emergency, which was also confirmed in the researched SAMU.

It is emphasized that pre-hospital care transfer requires the involvement of various organizations, teams, and professionals, creating a complex system that can result in specific challenges for high-quality transfer and, consequently, safe care^(14)^. The absence of standardized language in care transfer allows for a significant increase in adverse events^(15)^.

In this context, some protocols are used in an attempt to organize and expedite the process. Among them, we have: SBAR (Situation, Background, Assessment, and Recommendation); ATMIST (Name, Time of onset, Mechanism or complaint/history, Injury/investigations, Signs and Treatment); IMIST-AMBO (Identification, Mechanism/complaint, Injury/information related to the complaint, Signs and Symptoms, Treatment and Allergies, Medications, Brief History, and other relevant information); and ASCHICE (age, sex, history, injury, condition, estimated time of arrival)^(15)^. The absence of an implemented and trained protocol in MPHC services may contribute to information loss during patient transfer between pre-hospital and intra-hospital care systems. There is a need for standardization in patient transfer between institutions with the use of a transfer protocol^(16)^.

It is asserted that MIST (Mechanism of injury/illness, Injury sustained or suspected, Signs including observations, and Treatment given), IMIST-AMBO, and ATMIST are more comprehensive tools than SBAR, and should be the preferred choices for transferring information. This is because, besides aligning with SBAR, they provide more specific and accurate data pertaining to the patient’s clinical condition^(15,17)^.

In this sense, the information reported, either verbally or in writing, by the MPHC team will determine the continuity of care provided to the patient. Although there is an effort to implement a standard care transfer protocol, most recommendations do not address the structural, cultural, and professional contexts involved in the process. There is a need to invest in training, educational formation, and cultural change to promote safe and patient-centered care^(12,18)^.

Regarding medication safety, the teams administered medications only after prescription and followed the guidelines for medication administration recommended by the Brazilian National Health Surveillance Agency^(19)^.

In England and Wales, incidents related to the use of medications in mobile pre-hospital care were evaluated. Five ambulance care centers in these countries participated in the study. A total of 331 incidents involving 295 patients were found. Of these incidents, 166 (50.2%) were related to documentation errors, and 165 (48.2%) were related to clinical protocol errors regarding the dose to be administered to the patient^(19)^.

The peculiarities regarding the precariousness of care locations, management during unexpected actions, and the stress to which professionals are subjected make the whole chain of errors more likely to occur^(8)^. Failures in the preparation and administration of medications deserve attention for dosage errors, wrong medication, patient mix-ups, timing errors, wrong route, and documentation errors^(9,20)^. Therefore, it becomes essential to train the nursing team in basic medication administration procedures^(21)^.

Regarding the low adherence to hand hygiene by professionals in the researched Brazilian MPHC, improvements are needed, both in basic support and advanced life support teams, as hand hygiene during patient care has been shown to be a critical point. Supporting this statement, an Australian study pointed out that less than one-third of participants frequently sanitize their hands or follow global recommendations^(22)^. Among the difficulties encountered by Australian mobile pre-hospital care professionals, reports include: tension in care due to the patient’s severity; insufficient time for hand hygiene in contrast to the time needed for clinical care; delayed care resulting in warnings; lack of hand hygiene devices and products; skin reactions to alcohol-based products; and caring for two or more patients at once^(22)^.

The pre-hospital service professionals are not properly directed and trained in hand hygiene, as evidenced by the detection of a heavy bacterial load on the hands of, on average, 77% of the tracked professionals after patient care^(23)^. Regarding the use of gloves, Australian paramedics report that they use disposable gloves before patient care and change them when their integrity is compromised or when they become soiled. Except under such conditions, glove changes only occur at the end of a clinical case, with a single pair of gloves used throughout the care^(22)^.

When asked about handwashing, healthcare professionals in Portugal state that it is an important practice constantly followed in their care, where 90% of 50 observed nurses adhere to good hand hygiene practices. In a context where patient safety is a priority^(24)^, there are reflections on professional responsibility when not adhering to hand hygiene practices, considered imprudent and unethical. The first step for change is to develop facilitative means of accessibility to hand hygiene devices, with one of them being gel alcohol available outside the ambulance structure, presented in small bottles that can be attached to the team’s uniform. In parallel, regular glove changes after each procedure should be encouraged, along with the adoption of awareness-raising measures among the teams, such as printed reminders and alerts placed in the ambulances.

Patient falls are a frequent event with negative effects for all involved in the process^(25)^. There are several associated risk factors, among which orthostatic hypotension, arterial hypotension, arterial hypertension, bradycardia, psychomotor agitation, mental confusion, drowsiness, dizziness, seizures, hypoglycemia, among others^(26)^ stand out. Added to these are the conditions inherent to MPHC: areas of difficult access, presence of obstacles, exposure to weather, and urban violence. The research presented here observed a total of 12 incidents with falls, with 4 occurring in basic care and 8 in advanced care. It is noteworthy that the highest exposure to falls was related to patients in more serious health situations.

Studies demonstrate the importance of implementing preventive interventions to reduce the incidence of falls in hospitalized patients, such as: keeping bed rails/gurneys raised; providing guidance to those involved in the care process on fall risks and prevention; and keeping personal belongings close to the patient^(27)^.

### Study limitations

The study presented important limitations, such as data collection being conducted at a single center, which limits the generalizability of the findings, and being carried out by a single observer, which restricts comparisons between observations. Additionally, there is a scarcity of specific studies on patient safety in MPHC, both in the Brazilian and international contexts, which resulted in the use of references with topics closely related to those addressed and with a primary focus on intra-hospital care.

### Contributions to the healthcare field

The study is pioneering in Brazil by quantifying and presenting risks related to patient safety in MPHC. It highlights the need for investments in safety measures in MPHC, with the implementation of protocols and team training to mitigate healthcare risks in this setting. Thus, it can serve as a reference for the Brazilian Ministry of Health in revising its legislation on patient safety, which currently excludes the context of mobile pre-hospital care.

It is suggested that future studies investigate patient safety in MPHC in different scenarios than those presented here, aiming to provide relevant information for management and the implementation of strategies to maximize care improvement in this specific environment.

## CONCLUSIONS

Research on the safety environment offers an opportunity to identify areas that need improvement. The mobile pre-hospital care scenario is characterized as a generator of patient safety risks. The main risks related to international patient safety goals are related to communication between pre-hospital and intra-hospital teams, infection risks, and medication administration. Incidents related to the risk of falls are significant, requiring the implementation of preventive safety measures.

Strategies to intensify actions that lead to adherence to infection control measures, such as hand hygiene and regular glove changes, should be promoted through the use of active methodologies and team awareness measures.

Effective written communication can be a key element in maintaining patient safety, as it allows for the application of recording instruments, such as checklists and transfer protocols. The need for training and constant updates is imperative to ensure reliable, complete, and legible records, minimizing the loss of important information from patient care.
